# Squamous Cell Carcinoma of the Scalp Following Synthetic Hair Implantation: A Structured Narrative Review and the Seventh Case Reported in Literature

**DOI:** 10.7759/cureus.106938

**Published:** 2026-04-13

**Authors:** Leonardo Brambilla, Matilde Mantovani, Giorgio E Pajardi, Andrea Di Cristofori, Andrea Marchesi

**Affiliations:** 1 Plastic and Reconstructive Surgery, Fondazione IRCCS San Gerardo dei Tintori, Monza, ITA; 2 Plastic and Reconstructive Surgery, Università degli Studi di Milano, Milano, ITA; 3 Hand Surgery and Rehabilitation, Ospedale San Giuseppe, Milano, ITA; 4 Neurosurgery, Fondazione IRCCS San Gerardo dei Tintori, Monza, ITA

**Keywords:** artificial hair, latissimus dorsi flap, marjolin ulcer, scalp reconstruction, squamous cell carcinoma (scc), tdap

## Abstract

Synthetic hair implantation was previously employed as a cosmetic treatment for androgenic alopecia but has been associated with delayed complications related to chronic foreign body reactions.

We report a rare case of squamous cell carcinoma arising on the scalp at the site of prior synthetic hair implantation, many years after the initial procedure. The patient presented with a persistent, non-healing scalp lesion associated with recurrent inflammation and scarring in the area of previous implantation. Clinical evaluation and histopathological examination confirmed the diagnosis of squamous cell carcinoma, necessitating surgical management and reconstruction. This case draws attention to a potential long-term malignant complication of synthetic hair grafting and underscores the importance of maintaining a high index of suspicion when assessing chronic or recurrent scalp lesions in patients with a history of this procedure.

## Introduction

Synthetic hair implantation gained popularity in the 1970s and 1980s as a remedy for alopecia, primarily in men. The procedure of artificial hair implantation involved the use of acrylic and modacrylic fibers for hair replacement. This procedure offered advantages such as low cost and no need for donor site involvement. However, complications soon emerged, ranging from infection and chronic inflammation to pruritus and hair loss [1].

Despite being a rare complication, the development of malignant tumors such as squamous cell carcinoma (SCC) associated with synthetic hair implantation has been reported as a long-term complication, and the first case was described in 2012 [2].

Although no definitive causal relationship has been established, six documented cases of SCC following synthetic hair implantation have been reported in five case reports, underscoring the role of chronic inflammation in oncogenesis [2-6].

This review elucidates the pathogenesis of SCC in this context and examines the diverse reconstructive approaches employed after surgical excision. Furthermore, we present the seventh case report ever described. By contributing to the growing body of literature, this review aims to raise awareness about a rare but significant long-term complication of a once-popular cosmetic procedure. In doing so, we hope to facilitate earlier recognition and more effective management.

## Case presentation

**Figure 1:**
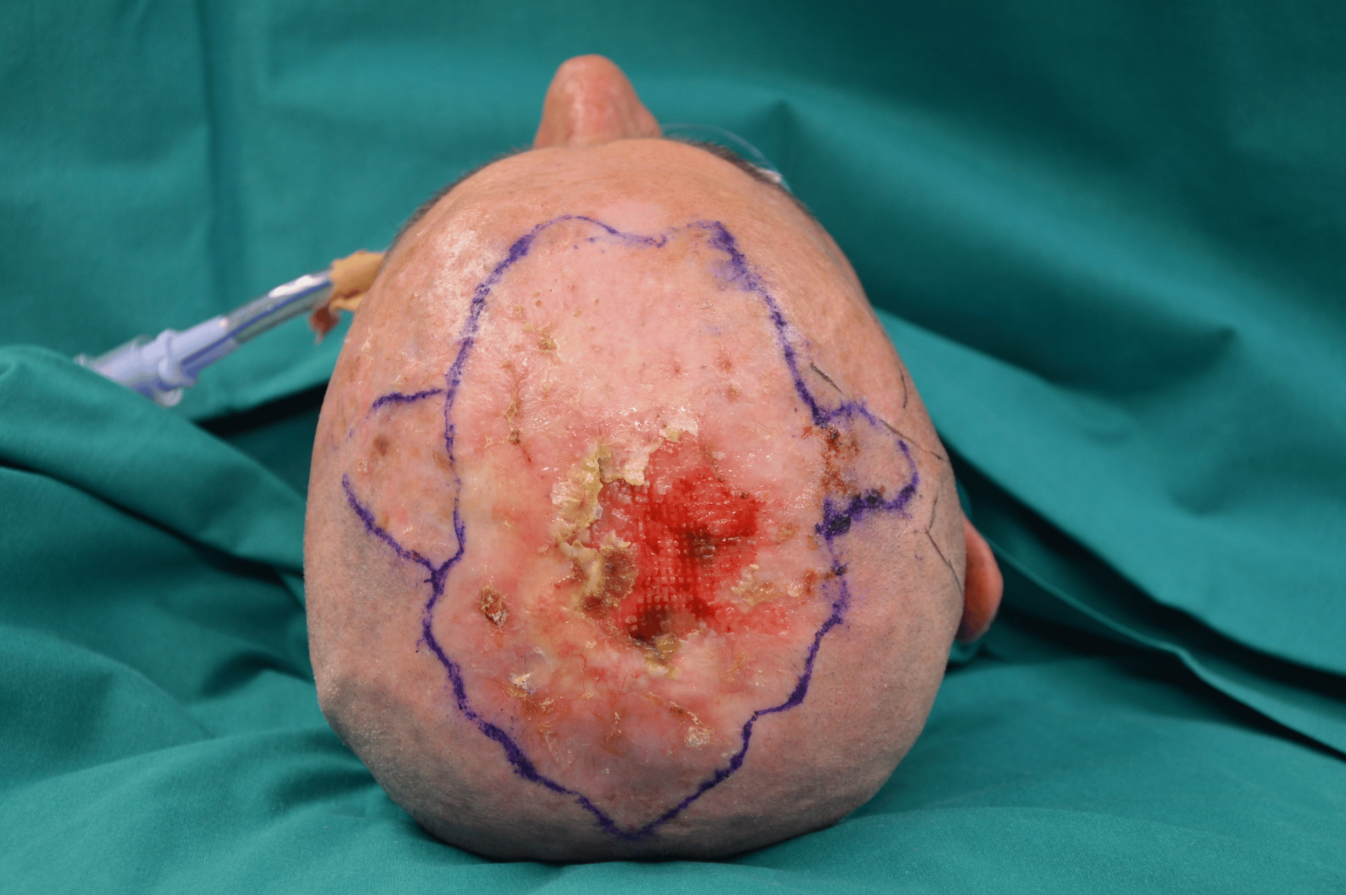
Median parietal head lesion. On examination, the lesion was ulcerated and friable, with spontaneous bleeding and malodor.

A 62-year-old male was referred to our hospital in 2023 for treatment of a median parietal skin lesion (Figure 1). The patient had undergone synthetic hair implantation on a regular basis for 17 years since the age of 32. 

He stated that the implantation was carried out using NIDO synthetic fibers composed of polyethylene. Typically, 250 to 400 fibers were implanted per session, and he reported a gradual reduction in the diameter of the artificial hair over time.

The patient sustained a traumatic laceration to the mid-parietal region in 2015. The resulting wound was managed with multiple dressings and healed through secondary intention at a different institution. However, toward the end of 2022, a new lesion reappeared at the same site, this time without any apparent trauma. It was a 4-cm nodular, ulcerated lesion within a 10-cm scar. An initial incisional biopsy, performed in February 2023, revealed actinic keratosis and colonization by methicillin-sensitive *Staphylococcus aureus*. After a course of antibiotic therapy, the lesion seemed to improve. However, in October 2023, a recurrence occurred with a new 2.5 cm ulcerated lesion. A second incisional biopsy, performed at our hospital, revealed the presence of an ulcerated and infiltrating SCC. Although no metastatic lesions were identified, the CT findings revealed a 3.6 mm depression of the cranial vault. Based on the staging studies, surgical excision of the lesion and reconstruction with a cranial prosthesis and a latissimus dorsi muscle free flap was planned.

**Figure 2:**
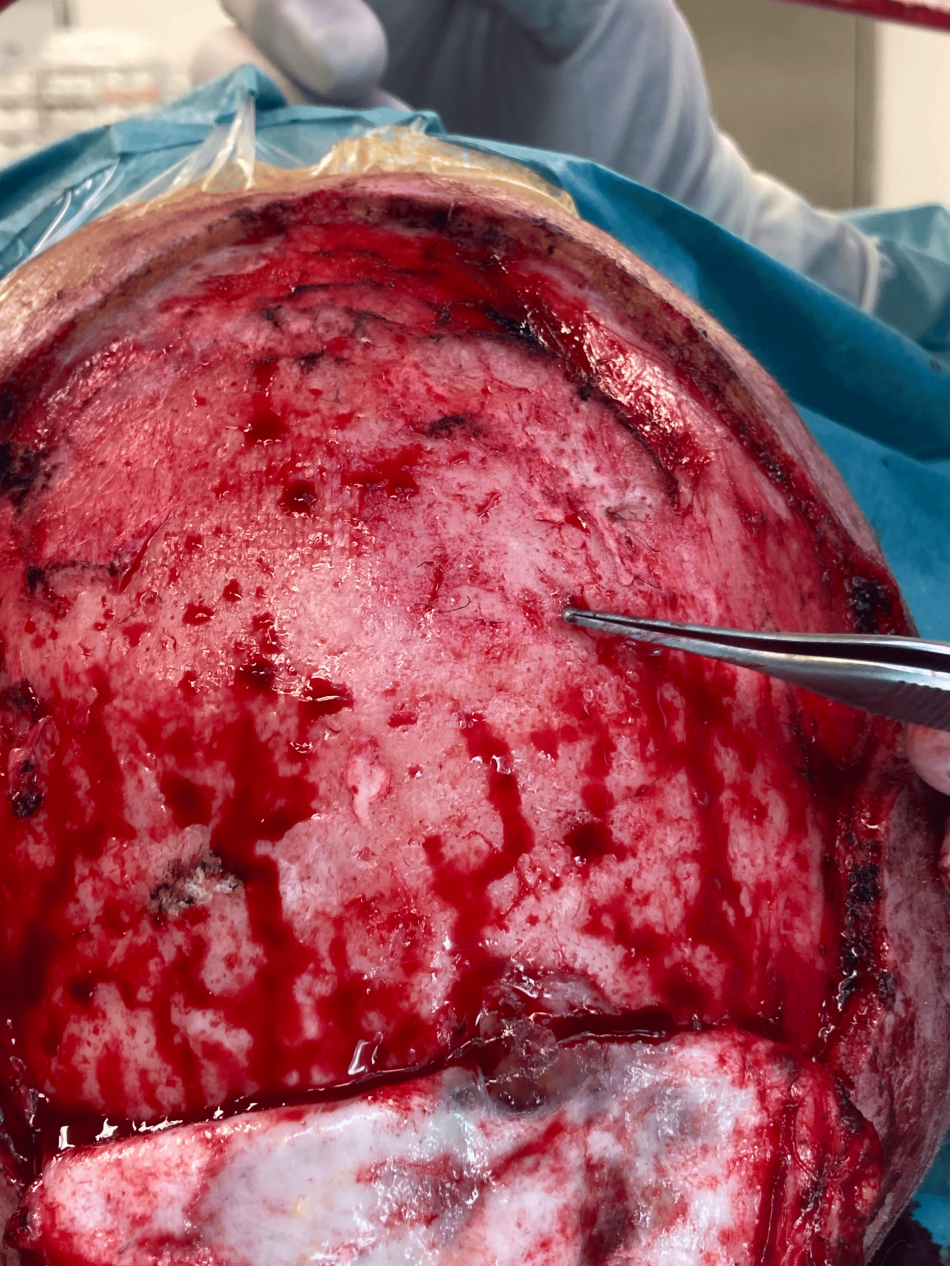
Skull exposed during surgery. Note the tumor infiltrating the cutaneous and subcutaneous tissue in the lower part.

**Figure 3:**
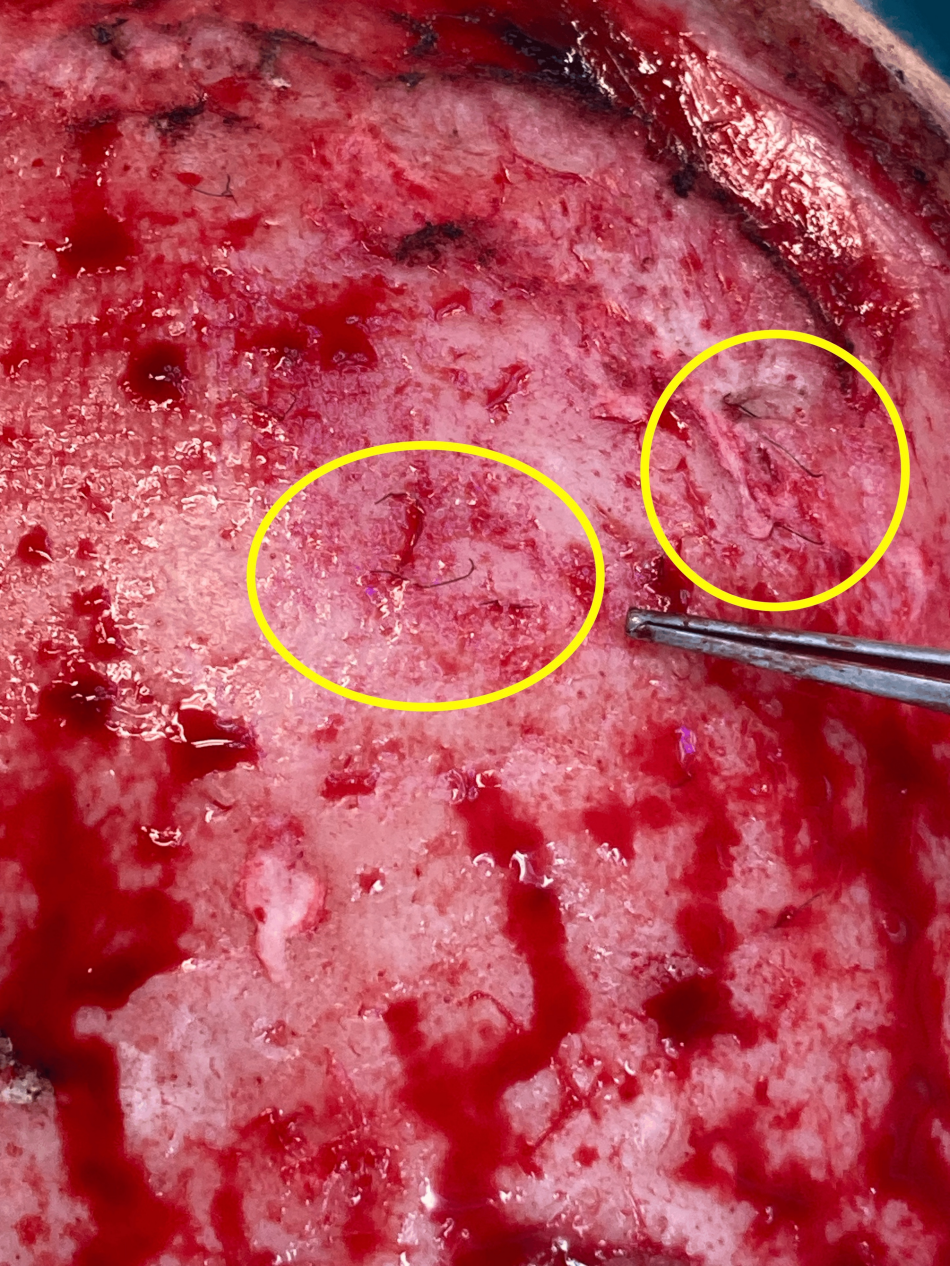
Circled in yellow: synthetic NIDO hair anchored to the skull

The surgery lasted 8 hours and included four stages. In the first stage, the patient was positioned in the left lateral decubitus. A muscle flap of latissimus dorsi was prepared, along with a thoracodorsal artery perforator (TDAP) skin island flap measuring 8x4 cm [7]. Microvascular dissection was performed on the thoracodorsal pedicle, which was isolated for approximately 10 cm. The flap was found to be viable at the end of the dissection and was then positioned at the level of the posterior axillary pillar. In the second stage, the patient was repositioned in the supine position. The known scalp lesion located on the parietal region, along with the surrounding scar tissue, was excised. The lesion contained synthetic prosthetic hair (Figures 2, 3), some of which were anchored to the cranial vault with associated cortical irregularities.

**Figure 4:**
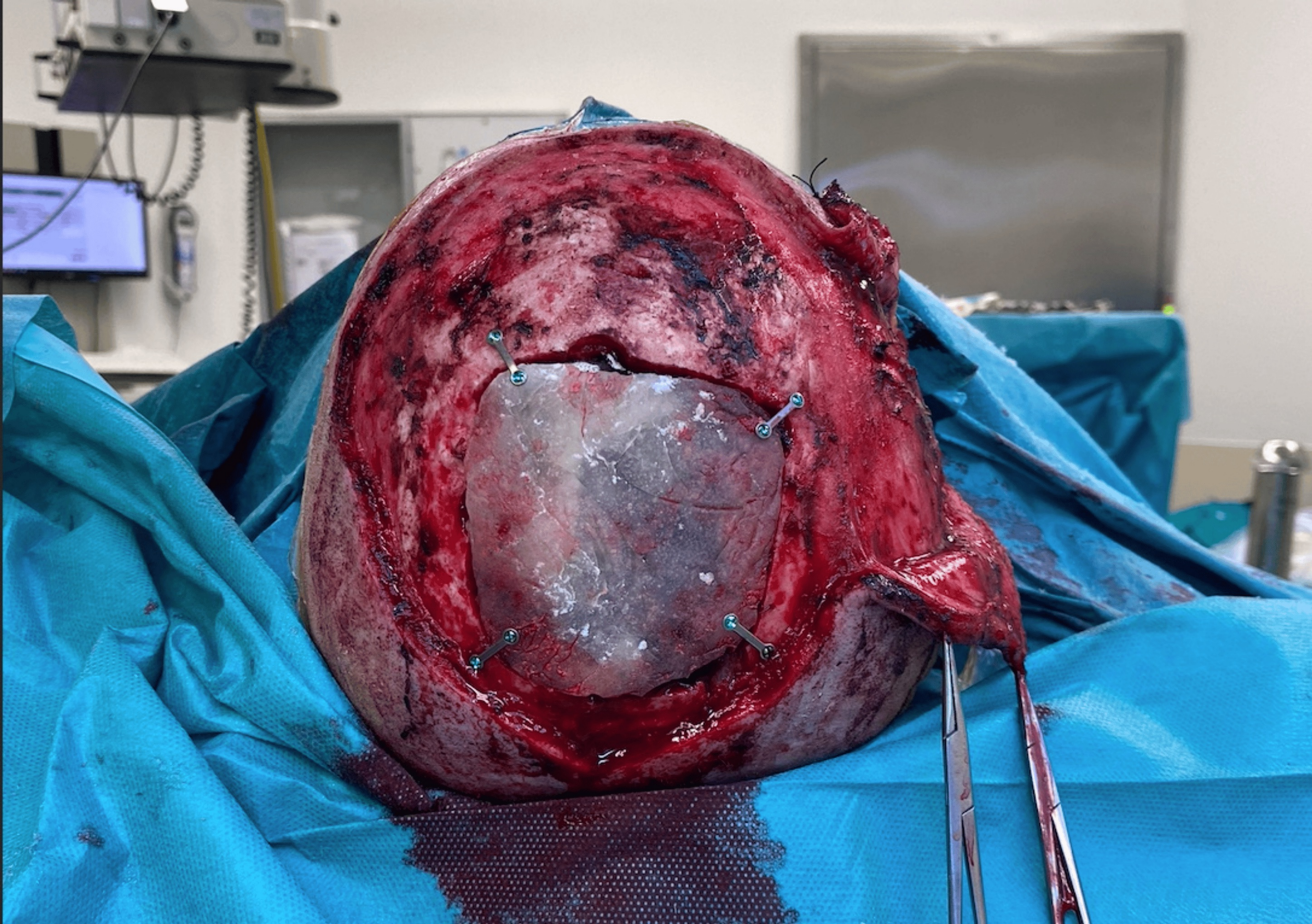
Poly-methyl-methacrylate prosthesis used during surgery

En bloc resection of the bone involved by the tumor was the first aim. During the neurosurgical stage, the bone involved by the tumor was identified and the healthy bone tissue. The area of bone to be resected was outlined, and five burr holes were created around it, followed by a craniotomy. No dural tears happened during the craniotomy. A poly-methyl-methacrylate prosthesis was used to seal the craniotomy, secured with four RM-compatible plates (Figure 4). 

**Figure 5:**
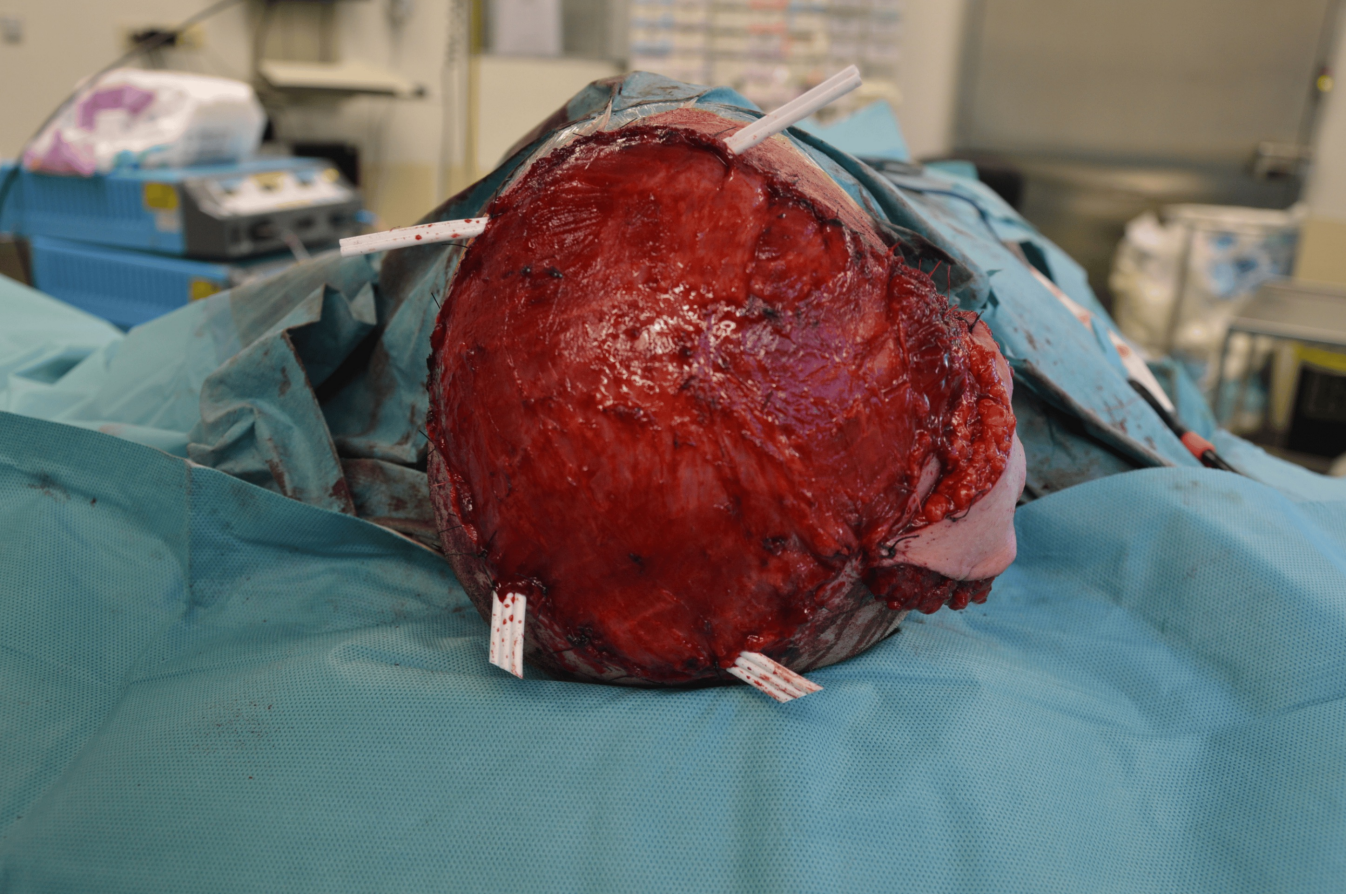
Superior view of the latissimus dorsi flap positioned to cover the defect before grafting, with laminar drains in situ, removed on postoperative day 3

**Figure 6:**
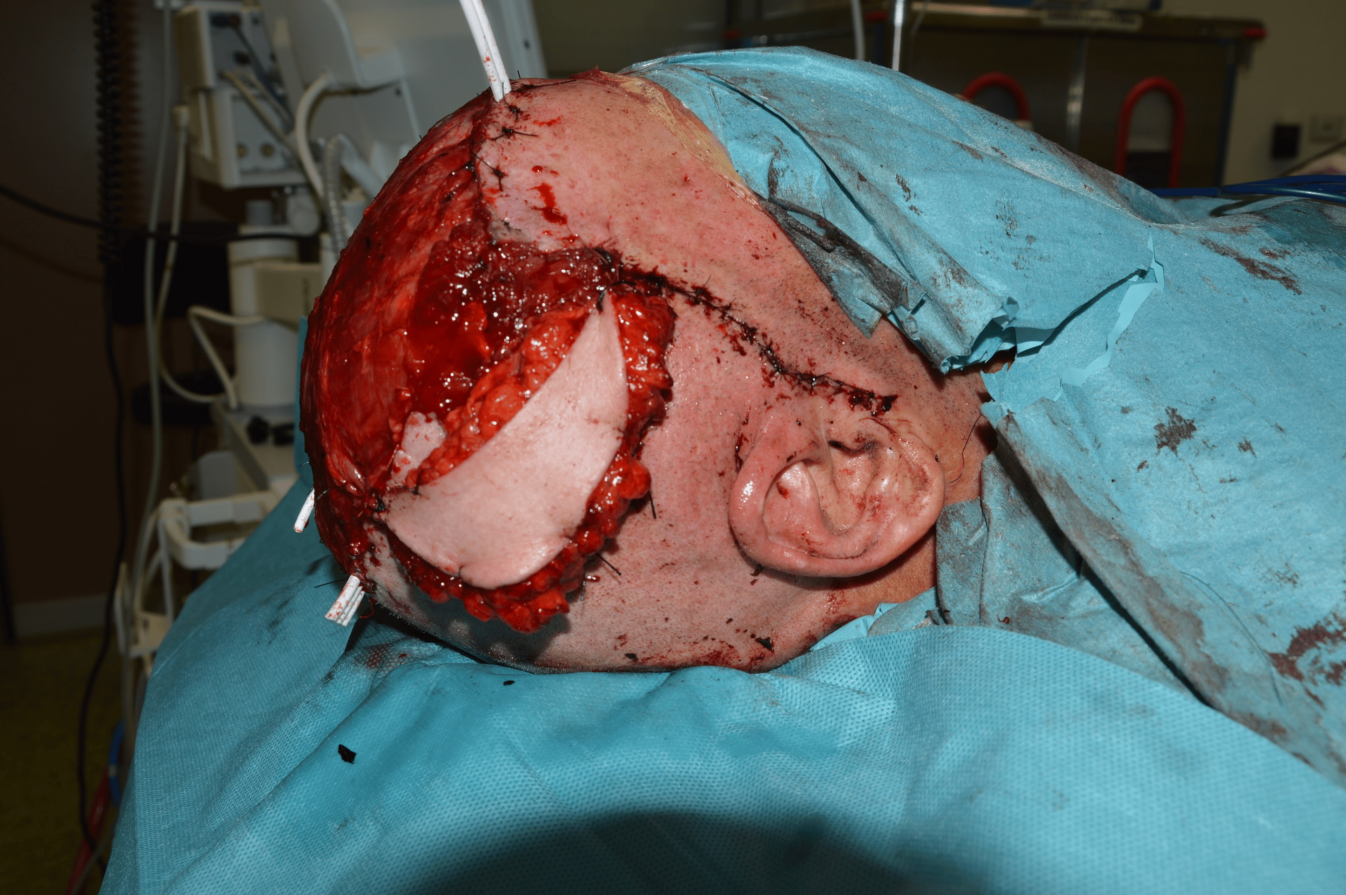
Lateral view showing the TDAP flap, which served as a vascular monitor; at the end of the surgical procedure, the flap appeared pink and well perfused. TDAP, thoracodorsal artery perforator.

During the last stage, the myocutaneous latissimus dorsi flap was used to cover the large surgical defect. Microsurgical anastomoses were made to the right temporal vessels. The skin island of a TDAP flap was used as a vascular monitoring site (Figures 5, 6) [8,9].

**Figure 7:**
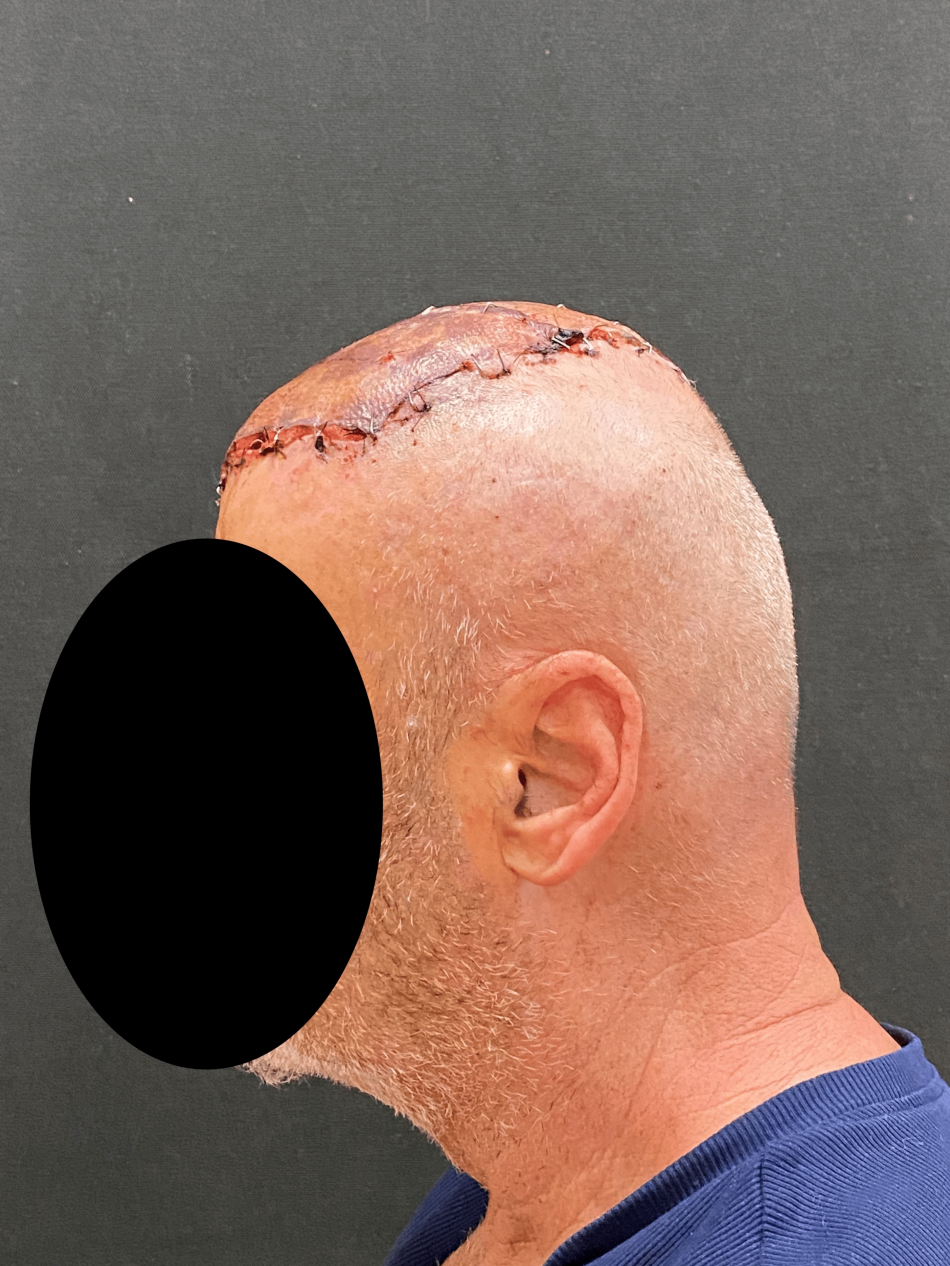
Lateral view of dermoepidermal grafts used to cover the free flap

**Figure 8:**
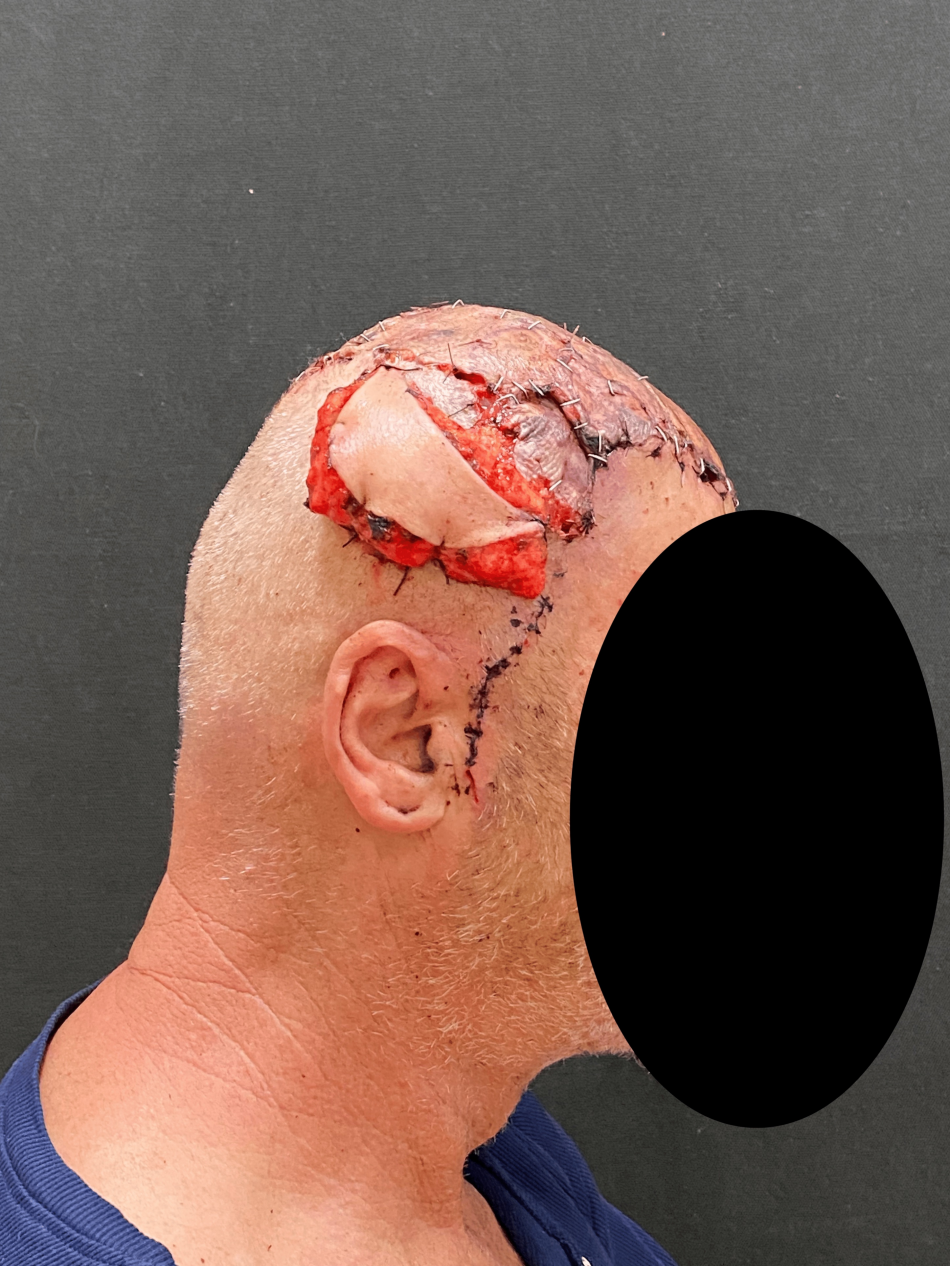
Lateral view of dermoepidermal grafts used to cover the free flap

**Figure 9:**
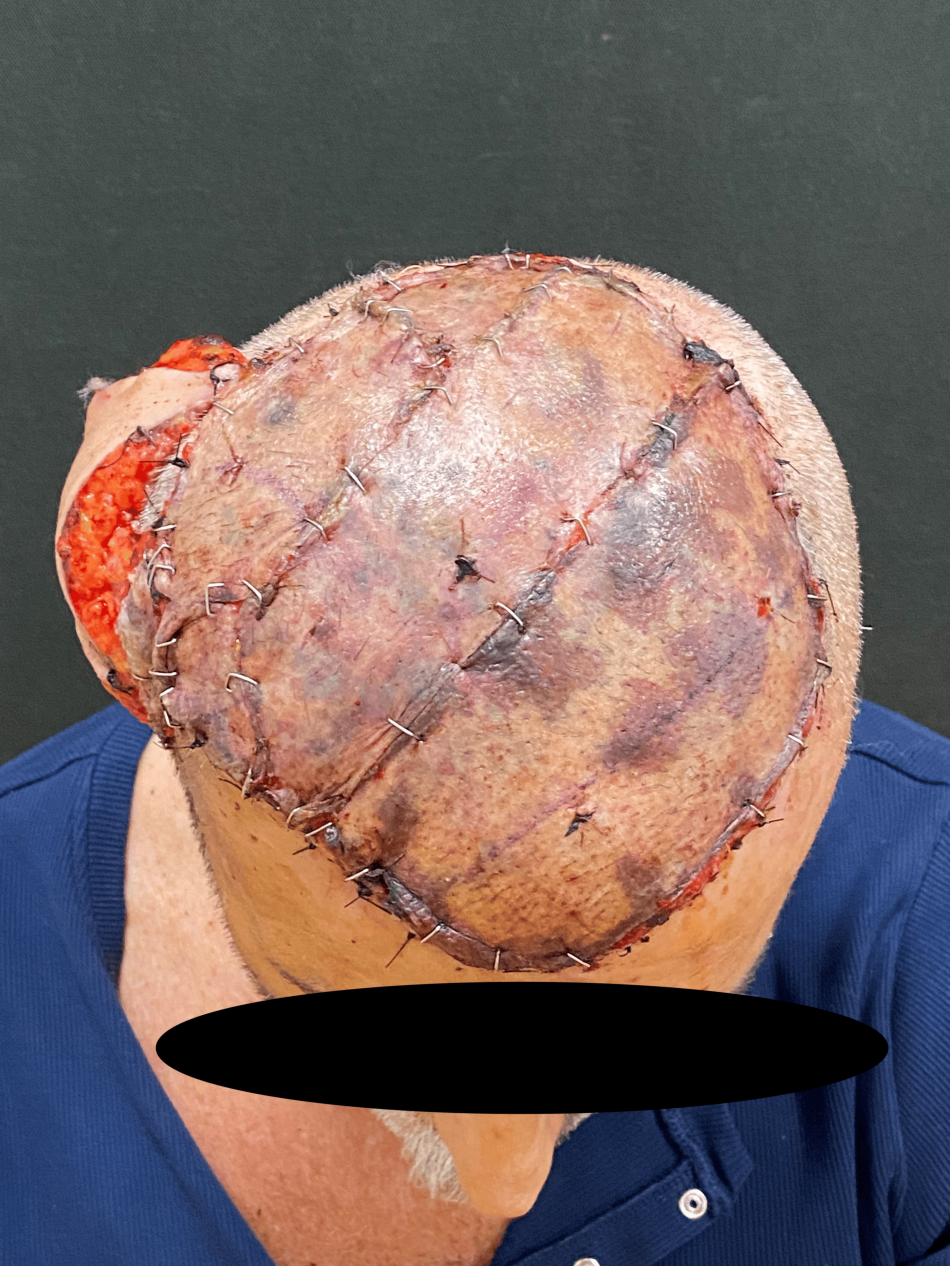
Superior view of dermoepidermal grafts used to cover the free flap

Partial-thickness dermoepidermal grafts were placed over the latissimus dorsi flap (Figures 7-9).

**Figure 10:**
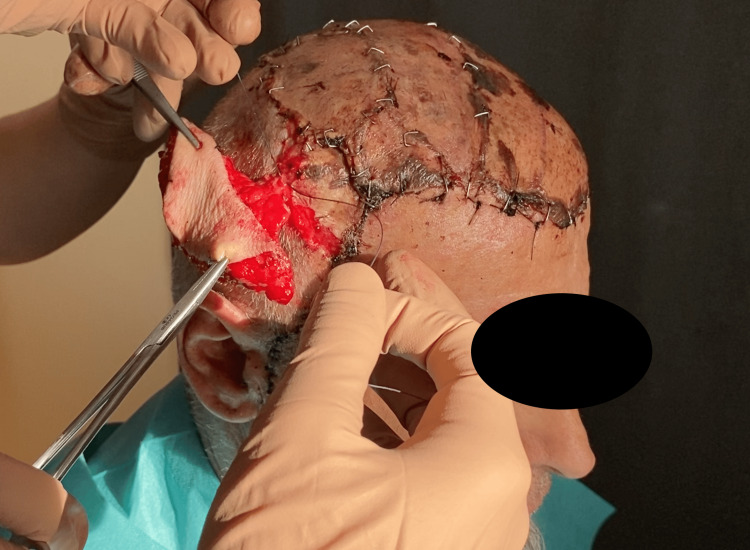
Ligation of TDAP flap TDAP, thoracodorsal artery perforator.

**Figure 11:**
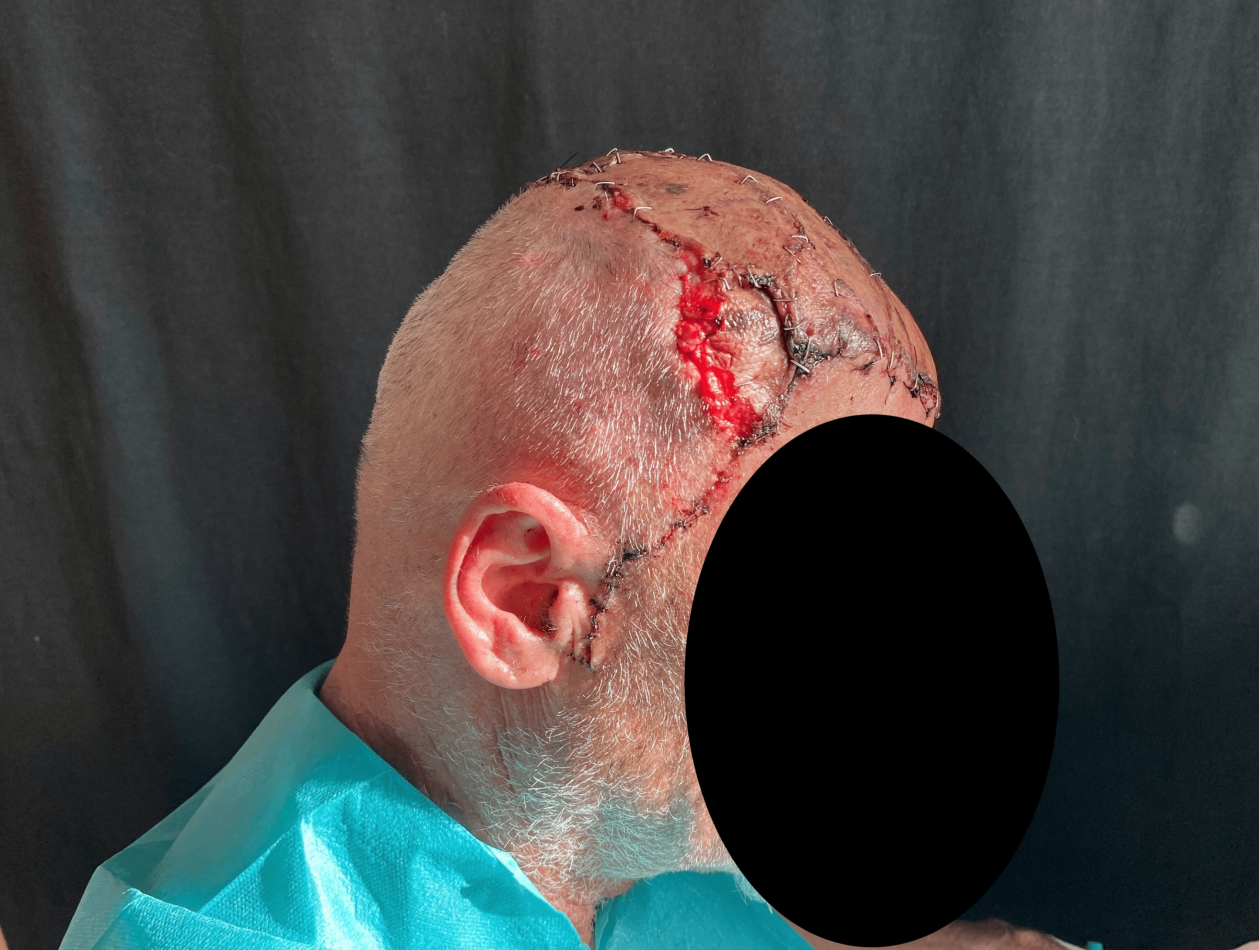
The patient just before being discharged home

The patient's hospitalization proceeded well. The skin island served as a reliable vascular monitor. Once flap stability was ensured, its ligation and resection were performed as a planned bedside procedure under sterile conditions at discharge, without the need for a second-stage operation in the main theater (Figures 10, 11).

**Figure 12:**
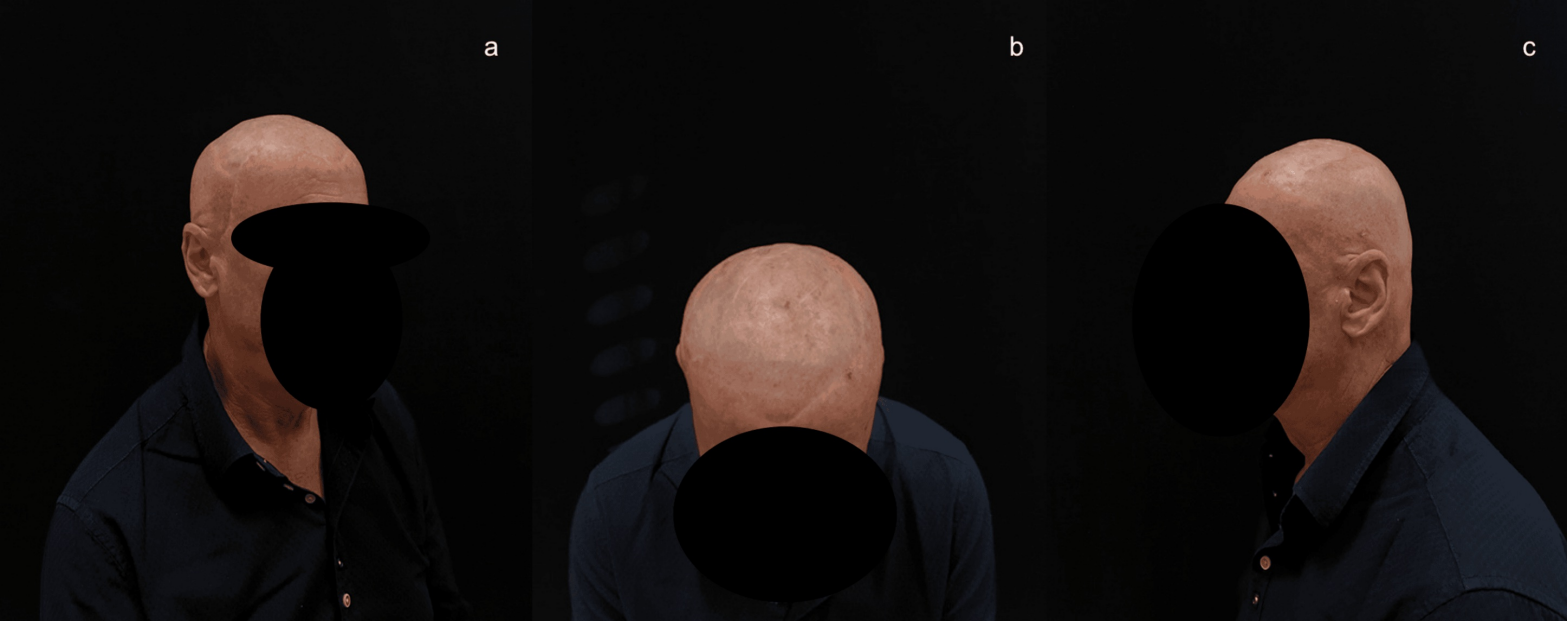
Pictures taken in December 2024. Different views of the cranium. Note that the flap has successfully healed, and only the suture margins are now visible.

Adjuvant radiotherapy consisting of 20 cycles was performed. Follow-up visits consistently showed excellent outcomes and no signs of disease relapse after 11 months (Figure 12).

## Discussion

Methods

This structured narrative review was conducted following a structured approach inspired by the Preferred Reporting Items for Systematic Reviews and Meta-Analyses (PRISMA) 2020 guidelines. 

A comprehensive literature search was performed to identify reports of SCC of the scalp associated with synthetic hair implantation. The following electronic databases were searched from inception to November 2025: PubMed/MEDLINE, Scopus, CINAHL, EMBASE/EMCARE, the Cochrane Library, and ResearchGate. The search strategy combined Medical Subject Headings (MeSH) terms and free-text keywords. The main search terms included: "squamous cell carcinoma", "scalp", "synthetic hair", "artificial hair implantation", "synthetic hair grafts", "synthetic fibers", "NIDO hair."

Boolean operators were applied as follows: ("squamous cell carcinoma" OR "SCC") AND ("synthetic hair" OR "artificial hair" OR "synthetic fibers" OR "NIDO hair") AND "scalp". Reference lists of all included articles were manually screened to identify additional relevant studies.

Inclusion criteria were: original articles or conference abstracts reporting cases of SCC of the scalp occurring at the site of synthetic hair implantation, human studies, and articles published in the English language.

Exclusion criteria were: studies not involving synthetic hair implantation, reports of non-SCC malignancies, reviews, editorials, commentaries, or animal studies without original patient data and duplicate publications.

**Figure 13:**
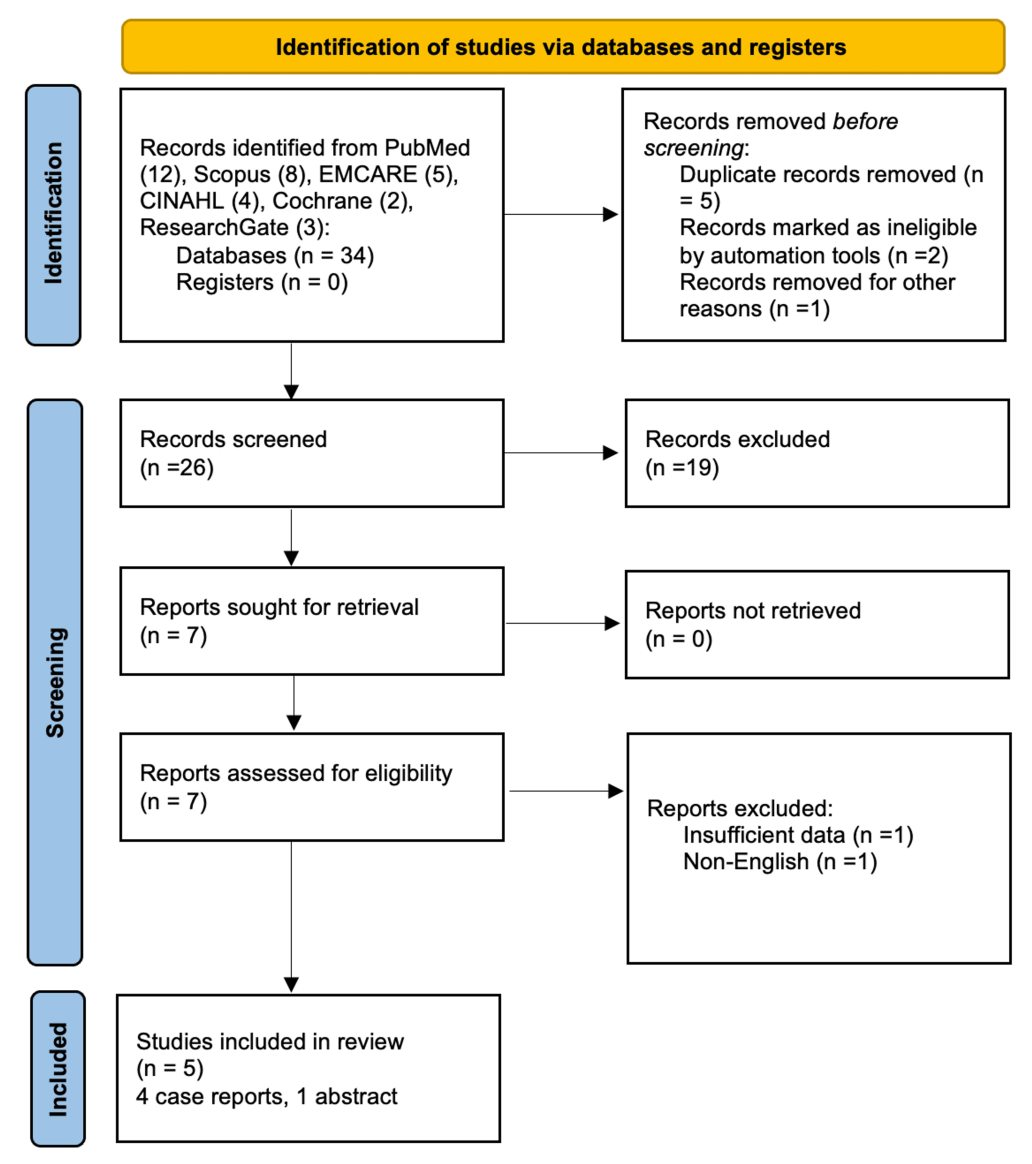
Preferred Reporting Items for Systematic Reviews and Meta-Analysis (PRISMA) flow diagram of the included studies

All records identified through database searching were imported into a reference manager, and duplicates were removed. Two authors independently screened titles and abstracts for relevance. Full-text articles were then assessed for eligibility. Discrepancies were resolved by consensus. From each included study, the following data were extracted: patient age, time interval between implantation and cancer diagnosis, histological grade, depth of tumor infiltration, reconstructive method, presence of metastasis, and duration of follow-up. Due to the rarity of the condition and the exclusive presence of case reports, a qualitative synthesis was performed rather than a meta-analysis. Figure 13 depicts the PRISMA flow diagram outlining the study selection and inclusion procedures.

Pathophysiology of SCC development

The carcinogenic potential of synthetic hair implantation has been primarily attributed to chronic inflammation, granulomatous reactions, and fibrosis caused by embedded fibers. Complications of synthetic hair implantation were first described in the late 1970s, with cases of infections, alopecia, and localized scarring. Lepaw examined 14 cases involving complications arising from the implantation of acrylic fibers into the scalp as a treatment for male-pattern baldness. In every instance, the complications were sufficiently serious to necessitate efforts to remove the fibers [1].

In 1983, the US Food and Drug Administration (FDA) prohibited this cosmetic procedure, followed by similar actions from health agencies in other countries, although the production and implantation of synthetic fibers continue on a small scale [10].

Early studies, such as those by Moizhess, demonstrated that foreign materials act as persistent irritants, creating an environment conducive to cellular dysplasia and malignancy [11].

Histological studies have revealed polyester or other synthetic fibers encapsulated in fibrotic tissue at the tumor sites. The hypothesis of a connection between prolonged fiber implantation and carcinogenesis was further suggested by Nicolas et al., who identified chronic inflammatory infiltrates and foreign body granulomas in SCC lesions [6].

Moreover, reports suggest that mechanical factors, such as poor maintenance of synthetic hair implants, further exacerbate tissue injury, amplifying the risk of malignant transformation. Atiyeh et al. and Shah et al. emphasized the role of such microtrauma in promoting epidermal hyperplasia and subsequent SCC development [5,12].

Previous cases in the literature 

Five case reports hypothesized a direct link between SCC and synthetic hair implants.

Although malignancy was not immediately recognized, in 2012, the first documented case of SCC of the scalp with bone involvement presumably linked to synthetic hair implants was reported [2]. The patient had undergone numerous hair implantation procedures over two decades and experienced repeated episodes of inflammation, infections, and folliculitis. These chronic conditions may have contributed to the development of the carcinoma, similar to the mechanism observed in Marjolin's ulcers. In this article, Chiarelli et al. emphasized the effectiveness of local flaps, such as rotational and transposition techniques, in addressing intermediate defects [2]. 

Nakayama et al. reported a case of a patient with a protruding tumor of 8 cm in diameter, corresponding to the artificial hair implantation area [3]. To cover the area of tumor removal, an anterolateral thigh (ALT) flap was harvested from the left thigh. The descending branch of the lateral femoral circumflex artery presented two primary perforators, allowing the design of a chimeric flap with two skin islands. These were repositioned in a circular arrangement to enable direct closure of the donor site. 

Maitani et al. discussed the third known case in English literature of SCC developing at the site of synthetic hair implants [4]. The patient, an 80-year-old man, presented with a large verrucous tumor located in the parietal region. A free latissimus dorsi flap was harvested from the left side of the back and used to cover an artificial bone structure within a scalp defect measuring 18 × 14 cm. The muscle flap was then covered using meshed split-thickness skin grafts.

Shah et al. presented an additional case attempting to link the occurrence of SCC to synthetic hair implants [5]. A deep inferior epigastric perforator flap has been employed for reconstructive purposes. Another report from Nicolas et al. described two different cases that appear to further support the hypothesis of SCC development potentially linked to synthetic hair implants, with most patients presenting advanced-stage tumors requiring aggressive surgical intervention. In both patients, an ALT free flap was elevated from the thigh and rearranged in a circular pattern to allow direct closure of the defect [6].

**Table 1:** Comparison of different key factors in seven case reports present in the literature ALT, anterolateral thigh; DIEP: deep inferior epigastric perforator; N/R: not reported; SCC: squamous cell carcinoma.

Study	Age (y.o.)	Time implant to cancer (years)	Histology	Infiltration level	Type of reconstruction	Metastasis	Follow-up (months)
Chiarelli et al., 2012 [2]	74	20	G1 SCC	Dura mater	Local flaps	No	6
Nakayama et al., 2021 [3]	48	18	G2 SCC	Cutaneous + subcutaneous tissue	Chimeric ALT	No	10
Maitani et al., 2022 [4]	80	28	G1 SCC	Dura mater	Latissimus dorsi	No	9
Shah et al., 2023 [5]	N/R	N/R	G2 SCC	pT4a dura mater + superior sagittal sinus	DIEP	No	18
Nicolas et al., 2025 [6]	57	15	N/R	Skull	ALT + fascia lata	No	N/R
Nicolas et al., 2025 [6]	72	9	N/R	Periosteum	ALT	No	N/R
Gatto et al., 2022 [7]	62	30	G2 SCC	Cutaneous + subcutaneous	Latissimus dorsi + TDAP vascular monitor	No	14

The data reported in Table 1 reveal significant variability in both patient age and tumor infiltration severity. Patients range from 48 to 80 years old, with one case where age is not reported. Most cases fall within the 60s and 70s, suggesting that SCC associated with implants takes decades to develop. The youngest reported patient, at 48 years old, exhibited only cutaneous and subcutaneous infiltration, whereas older patients, such as the 80-year-old case, showed deeper invasion into the dura mater.

For the seven cases identified, the time elapsed between implantation and cancer development was reported in six instances. The median interval was 19 years, with values ranging from 9 to 30 years, highlighting a long latency period. This suggests that malignancies associated with implants may develop over decades, reinforcing the need for long-term follow-up in patients with implants. In our case, the initial diagnosis of actinic keratosis was based on a biopsy performed at an external center. The subsequent finding of infiltrating SCC only a few months later suggests significant lesion heterogeneity or a potential sampling error in the first instance, although rapid clinical progression must also be considered as a contributing factor.

A striking observation in the data is that none of the reported cases developed metastasis, despite significant variability in infiltration severity. Even in cases where the tumor had invaded deep structures such as the dura mater, skull, and superior sagittal sinus, there was no evidence of distant spread. This suggests that implant-associated SCC may primarily exhibit localized aggressive behavior rather than a high propensity for metastasis. The relatively short follow-up periods, ranging from 6 to 18 months, could also mean that metastasis had not yet had time to develop or be detected. Longer-term studies would be necessary to determine whether metastasis could emerge beyond the observed follow-up windows.

The evidence reviewed in this study highlights a plausible link between synthetic hair implantation and the development of SCC. Although the relationship remains speculative due to the limited number of cases, chronic inflammation and foreign body reactions appear to play a central role. Persistent irritation caused by synthetic fibers embedded within the scalp tissue likely fosters a microenvironment conducive to malignancy. This phenomenon mirrors mechanisms observed in Marjolin’s ulcers, where chronic wounds and inflammation lead to cellular dysplasia and eventual carcinoma.

In the context of synthetic hair implantation, the continuous exposure to foreign materials, combined with recurrent infections and microtrauma, exacerbates tissue damage. Histopathological studies reinforce this hypothesis, revealing the presence of foreign body granulomas, fibrosis, and inflammatory infiltrates in SCC lesions associated with implanted fibers. These cases underline the importance of long-term vigilance in individuals with a history of synthetic hair implantation, as the risk of malignancy may manifest decades after the procedure.

Our case represents the seventh documented instance of SCC linked to synthetic hair implants and highlights the multifaceted challenges associated with its diagnosis and treatment. The involvement of the cranial vault in this case necessitated a multidisciplinary approach, incorporating oncological, neurosurgical, and reconstructive expertise. The innovative use of the latissimus dorsi flap in conjunction with the TDAP flap as a sentinel monitoring tool ensured optimal visualization of perfusion and minimized the need for invasive monitoring techniques. 

## Conclusions

Chronic inflammation is a potential mechanism driving SCC development in synthetic hair implantation. Clinicians should maintain a high index of suspicion for malignancy in patients presenting with chronic inflammation or recurrent lesions at sites of synthetic hair implantation. Long-term vigilance in individuals with a history of synthetic hair implantation is recommended, as the risk of malignancy may manifest decades after the procedure.

Future research should focus on larger retrospective studies and collaborative registries to better elucidate the relationship between synthetic hair implantation and SCC.
